# Unveiling the Truth in Pain: Neural and Behavioral Distinctions Between Genuine and Deceptive Pain

**DOI:** 10.3390/brainsci15020185

**Published:** 2025-02-12

**Authors:** Vanessa Zanelli, Fausta Lui, Claudia Casadio, Francesco Ricci, Omar Carpentiero, Daniela Ballotta, Marianna Ambrosecchia, Martina Ardizzi, Vittorio Gallese, Carlo Adolfo Porro, Francesca Benuzzi

**Affiliations:** 1 Department of Biomedical, Metabolic and Neural Sciences, University of Modena and Reggio Emilia, 41125 Modena, Italy; vanessa.zanelli@unimore.it (V.Z.); claudia.casadio@unimore.it (C.C.); francesco.ricci@unimore.it (F.R.); 269868@studenti.unimore.it (O.C.); daniela.ballotta@unimore.it (D.B.); carlo.porro@unimore.it (C.A.P.); francesca.benuzzi@unimore.it (F.B.); 2 Neuroscience Unit, Department of Medicine and Surgery, University of Parma, 43125 Parma, Italy; marianna.ambrosecchia@gmail.com (M.A.); martina.ardizzi@unipr.it (M.A.); vittorio.gallese@unipr.it (V.G.); 3 Center for Studies and Research in Cognitive Neuroscience of Cesena, 47522 Cesena, Italy

**Keywords:** pain, facial expressions of pain, deception, cingulate cortex, fMRI

## Abstract

**Background/Objectives**: Fake pain expressions are more intense, prolonged, and include non-pain-related actions compared to genuine ones. Despite these differences, individuals struggle to detect deception in direct tasks (i.e., when asked to detect liars). Regarding neural correlates, while pain observation has been extensively studied, little is known about the neural distinctions between processing genuine, fake, and suppressed pain facial expressions. This study seeks to address this gap using authentic pain stimuli and an implicit emotional processing task. **Methods**: Twenty-four healthy women underwent an fMRI study, during which they were instructed to complete an implicit gender discrimination task. Stimuli were video clips showing genuine, fake, suppressed pain, and neutral facial expressions. After the scanning session, participants reviewed the stimuli and rated them indirectly according to the intensity of the facial expression (IE) and the intensity of the pain (IP). **Results**: Mean scores of IE and IP were significantly different for each category. A greater BOLD response for the observation of genuine pain compared to fake pain was observed in the pregenual anterior cingulate cortex (pACC). A parametric analysis showed a correlation between brain activity in the mid-cingulate cortex (aMCC) and the IP ratings. **Conclusions**: Higher IP ratings for genuine pain expressions and higher IE ratings for fake ones suggest that participants were indirectly able to recognize authenticity in facial expressions. At the neural level, pACC and aMCC appear to be involved in unveiling the genuine vs. fake pain and in coding the intensity of the perceived pain, respectively.

## 1. Introduction

The International Association for the Study of Pain (IASP) defines pain as an unpleasant sensory and emotional experience associated with, or resembling that associated with, actual or potential tissue damage [1]. This dual sensory-emotional nature makes pain not only a sensory phenomenon but also a crucial social signal, often expressed through specific facial movements.

Facial expressions serve as universal signals of human emotions and can reliably communicate internal states across different contexts in highly social species [2,3]. Pain, as a complex emotional and sensory experience, is accompanied by distinctive facial expressions, which facilitate social communication and support-seeking behaviors by eliciting empathy [4,5,6]. Empathy is broadly defined as the capacity to understand what others feel, whether it is an emotional or sensory state [7]. In literature, human pain-related body behaviors—especially noticeable actions such as limping or guarding—have been found to influence social reinforcement and social behavior. Interestingly, less attention has been given to facial expressions of pain [8]. The literature on pain expression processing and empathy has mostly relied on stimuli featuring body parts rather than faces [9,10]. Indeed, facial pain expressions have a special survival and communicative value due to the fact that they can warn others of imminent danger and elicit helping behavior [4,11]. A typical pain-related facial expression tends to appear consistently across different pain conditions, making it universally recognizable [6]. This set of facial movements include lowered brow, raised cheeks, tightened eyelids, an opened mouth, and a wrinkled nose [6,12]. These features are distinctive of pain and can be differentiated from expressions of other negative emotions such as disgust, fear, or anger [12].

Nevertheless, emotions expressed through nonverbal cues do not always align with an individual’s actual emotional state [13] because of the evolutionary development of interpersonal deception [2,3,11,14,15]. Deception is defined as “an act that is intended to foster in another person a belief or understanding which the deceiver considers false” [16]. This ability must be intentional and conscious, meaning that lying inherently reflects human intention and awareness [14]. There are three major ways in which emotional expressions could be intentionally manipulated. First, an expression may be simulated; namely, individuals display an emotion (e.g., pain) they are not genuinely experiencing. Secondly, an expression can be masked by replacing the genuine emotion with an artificial emotional display (e.g., the person is truly experiencing sadness but displays a fake smile). Finally, a facial expression can be suppressed in such a way as to conceal the emotion the individual is experiencing (e.g., the person is experiencing pain but inhibits the facial expression to conceal it) [15]. According to this distinction, both simulated (or fake) and suppressed expressions are clear examples of deception.

In the past few decades, several attempts have been made to determine the distinctive morphological/visual patterns, both static and dynamic, of deceptive facial expressions compared to genuine ones [17]. In general, spontaneous emotional expressions are more symmetrical compared to those made deliberately, both for positive and negative emotions [2]. Moreover, genuine expressions usually last between 2 and 4 s, whereas deliberate expressions show abrupt onset and offset, and the apex lasts too long [2]. More specifically, simulated (or fake) pain expressions exaggerate typical pain features, such as brow lowering and mouth opening, in both intensity and duration, and include non-pain-related emotions (e.g., shame, happiness; [12,15]). On the other hand, suppressed pain facial expressions are less intense and show residual facial activity due to the pain actually experienced [12].

While the distinctions between simulated and genuine facial expressions of pain might appear evident from the literature, it may be surprising to learn that individuals are not particularly skilled at detecting deception. Actually, several studies have demonstrated that when individuals are asked whether a person is deceiving or not (i.e., direct tasks), their performance is slightly above chance, even if they undergo recognition training [3,15,18,19]. A possible explanation is that people consciously tend to rely on cues, such as brow lowering, likely because of their salience in mental representations of pain expressions, which, however, may not be the best predictors of real pain [6]. Instead, according to some authors, the recognition of pain from facial expressions should depend on other prominent facial cues (eye narrowing [3,6]).

Interestingly, using some kind of indirect tasks (e.g., asking to rate the perceived cooperativeness; [20]), subjects are better at recognizing when emotions are simulated. In line with this view, a study by Stel and van Dijk [13] demonstrated that participants were good at detecting liars when using an indirect task, i.e., to indicate the extent to which the target was experiencing a particular emotion while telling a story, compared to a direct task in which they rated the truthfulness of the target.

While in the field of deception research, the terms ‘indirect’ and ‘direct’ are specifically defined as outlined previously, in the field of emotion research, authors use the terms ‘explicit’ and ‘implicit’ to make a distinction between different tasks. Explicit emotional processing requires participants to label the affective content of stimuli or to perform a valence categorization task (e.g., determining whether a stimulus is positive or negative). It needs declarative evaluation and involves higher cognitive resources to define conscious emotional states. On the other hand, implicit tasks consist of asking the participant to process a non-emotional attribute of the stimulus (e.g., gender discrimination task). This process is meant to be automatic and does not require conscious access to be executed [21,22], and it is based on the assumption that emotional processes operate independently of cognitive attention [23].

Concerning the neural correlates of pain, two distinguishable sets of areas are involved in the sensory and affective components of pain, also collectively referred to as the “pain matrix” [24,25,26]. The primary and secondary somatosensory cortices (S1, S2) and the posterior insula process localization, quality, and intensity discrimination of painful stimuli (i.e., sensory components; [27]). Conversely, the anterior mid-cingulate cortex (aMCC; see [28] for an updated nomenclature of cingulate cortex) and the anterior insula (AI) are engaged in processing the distressing aspects of pain, including the subjective discomfort associated with the nociceptive signal and the motivational drive to eliminate the stimulus responsible for this experience (i.e., affective components; [29]), and their activity correlates with the intensity of perceived pain [30].

These “affective” areas have been found to be activated also during the observation of facial expressions of pain [5,31,32]. However, relatively few studies have focused on the neural mechanisms underlying the distinctions between genuine and fake facial expressions of pain. For example, Zhao and colleagues [33] demonstrated some neural differences between the observation of genuine and fake facial expressions of pain. However, in that study, participants were explicitly informed that they were observing simulated expressions; moreover, even “real” pain expressions were actually simulated by actors.

The aim of this study is to investigate the differences in neural correlates underlying the observation of genuine, fake, and suppressed pain facial expressions. In other words, we seek to determine whether the brain can differentiate between genuine and deceptive expressions. To the best of our knowledge, this is the first experimental design about genuine/deceptive pain that exclusively used video clips showing only facial expressions and not injured body parts, with genuine and suppressed pain videos derived from real painful stimuli applied to the models’ hands, thus ensuring the authenticity of the emotional displays. Moreover, during functional magnetic resonance imaging (fMRI) scanning, participants performed an implicit task (gender discrimination), remaining unaware of which videos involved genuine pain. Additionally, participants later reviewed the videos outside the scanner to provide indirect ratings (e.g., perceived intensity of the expression and of the pain), enabling us to assess whether they could indirectly differentiate between genuine, simulated, and suppressed pain.

## 2. Materials and Methods

### 2.1. Participants

Twenty-four right-handed healthy women (mean age = 20.7 years, SD ± 2.9; school age = 13.1 years, SD ± 0.6) were recruited among the students of the University of Modena and Reggio Emilia to take part in the experiment. Handedness was assessed using the Edinburgh Inventory (M = 0.9, SD ± 0.1; [34]). The volunteers had no history of neurological or psychiatric diseases or brain injury, and they had no exclusion criteria for MR. Because of the differences between genders both in expressivity [35,36] and in empathic behavior [37,38], we decided to recruit only women in order to have a homogenous experimental population to study. All participants gave their written informed consent to take part in the study, and they received study credits as a fee for their attendance. The experiment was previously approved by the Local Ethics Committee (Area Vasta Emilia Nord, protocol number 134.14), and it was conducted in accordance with the ethical standards of the 2013 Declaration of Helsinki [39].

### 2.2. Questionnaires and Interoception Assessment

Before beginning the fMRI experimental procedure, participants completed a series of self-administered questionnaires in their Italian printed version:

- Empathy Quotient (EQ; [40]);
- Interpersonal Reactivity Index (IRI; [41]);
- Toronto Alexithymia Scale 20 (TAS-20; [42]);
- Pain Catastrophizing Scale (PCS; [43]).

Moreover, we decided to investigate interoceptive accuracy (IA), namely, the individual sensitivity to physiological stimuli originating inside the body, which is one of the most relevant aspects of self-experience and may influence the perception and evaluation of emotional stimuli [44,45]. IA can be assessed through a heartbeat perception task [46], and in the present study, this task was employed as described in Ardizzi et al. [47] and Ambrosecchia et al. [48]. Briefly, the task consisted of silently counting one’s own heartbeats while ECG was being recorded, without any kind of feedback from the experimenters. After a 15 s training period, participants had to count their heartbeats in four different randomized time intervals (25 s, 35 s, 45 s, and 100 s), which were triggered by audio-visual start and stop cues. ECG was recorded through three Ag/AgCl, pre-gelled, 10 mm electrodes (ADInstruments, Oxford, UK) placed in an Einthoven’s triangle configuration, with a sample size of 1 KHz and with an online filter. Afterwards, the R-wave peaks were detected in order to obtain the real number of the participants’ heartbeats (Powerlab and OctalBioAmp 8/30, ADInstruments, United Kingdom).

The IA score was calculated as the mean score of the four separate heartbeat perception intervals according to the following transformation [46,49]:

$$\frac{1}{4}\sum (1-\frac{\left(\left|recorded\;beats-counted\;beats\right|\right)}{recorded\;beats})$$

According to this transformation, the IA score varies between 0 and 1, with higher scores indicating smaller differences between objectively recorded and subjectively counted heartbeats (i.e., higher interoceptive accuracy).

### 2.3. Experimental Procedure

**Figure 1:**
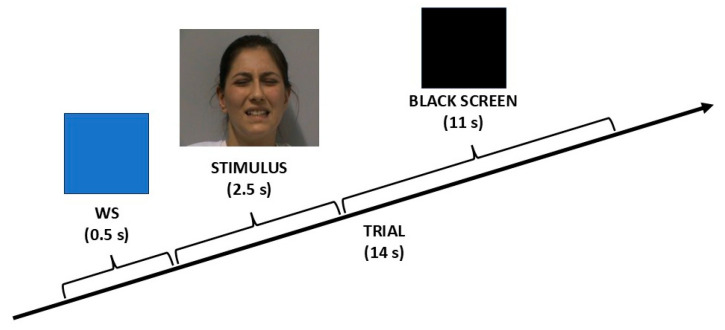
Experimental design. Each trial (14 s) was composed of these e a brief warning signal (WS) of 0.5 s, video clip presentation (2.5 s), and a continuous black screen (11 s) until the next trial.

An event-related fMRI paradigm was employed. Each participant completed three runs of 36 trials (for a total of 108 trials). Every trial lasted 14 s and began with a brief visual warning signal (WS), which was a change in the background color of the video from black to blue (0.5 s). Then, the WS was followed by stimulus presentation (2.5 s) and a black screen (11 s; see Figure 1). At the beginning and at the end of each run, there was a 20 s rest period.

The stimuli were 27 video clips (20 female and 7 male identities) that belonged to different categories: genuine pain (GP; video clips reproducing facial expressions of individuals who were truly experiencing pain in their hand); deceptive pain, comprising fake pain (FP; video clips representing face expressions of individuals who were asked to simulate a painful experience) and suppressed pain (SP; video clips depicting individuals who were really experiencing pain in their hand but who were asked to suppress their facial expressions); neutral (N; these video clips were used as the control condition and depicted individuals with a neutral expression while receiving a light touch on their hand).

Starting from the 108 video clips, we created four different randomized sequences to present to the participants. Each sequence consisted of 36 video clips per run, divided into 9 GP, 9 FP, 9 SP, and 9 N.

Stimuli were presented via the Esys-fMRI (Invivo Corporation, Gainesville, FL, USA) remote display, timed by custom-made software developed in Visual Basic 6 (http://digilander.libero.it/marco_serafini/stimoli_video/, accessed on 8 January 2015).

Participants were instructed to complete an implicit task, i.e., they had to carefully watch the video and press a button on a response box (Current Designs Inc., Philadelphia, PA, USA) when they detected a male identity in the video clip.

At the end of the scanning session, each volunteer was asked to review the same video clips on a computer outside the magnetic resonance room and to give *a posteriori* ratings. Using a range from zero to ten (with 0 = no intensity and 10 = the maximum imaginable intensity), they had to rate two indirect parameters: the intensity of the facial expression (IE) and the intensity of the pain they guessed had been really perceived by the individual shown in each video (IP). When participants considered a certain video clip to be expressive but not a facial expression of pain (IE ≠ 0, IP = 0), they were also asked to indicate which emotion they believed it represented.

### 2.4. Stimuli

The video clips employed as stimuli were recorded and validated in a previous experiment by our research group [5]. Twenty-seven young participants were recorded while experiencing painful or non-painful stimulations on their right hand. They sat comfortably in front of a grey background, wearing a white coat covering their personal clothing, without any kind of distinctive elements (e.g., make-up or jewelry). The experimenter delivered the painful or tactile stimulation manually through an aluminum hollow cylinder containing a sliding brass weight connected to a plastic tip. This tip could sustain either a stainless-steel wire (0.2 mm section) or a foam rubber tip (2 mm). In a preliminary calibration procedure, the brass weight was set so that the subject consistently reported no pain when touched with the foam rubber tip (pain intensity = 0 on a numerical rating scale–NRS–from 0 to 10) and reported a light-moderate pain when touched with the wire (pain intensity = 2–4 on the same 0–10 NRS). In each of the first two sessions, 20 painful and 20 non-painful stimuli were pseudo-randomly alternated: in the first session, the volunteers were instructed to react naturally to each stimulus (GP and N video clips); in the second session, they were instructed to suppress pain during actual painful stimulation (SP and, again, N video clips). After each stimulation, the subject reported the perceived pain intensity on the 0–10 NRS. Finally, in a third session, participants did not undergo any stimulation, but they were asked to simulate some painful experiences (FP video clips). The camera (Sony HDD handycam DCR-SR32, spatial resolution 720 × 576 pixels; Sony, Tokyo, Japan) was arranged 1.5 m in front of the individuals at eye level. The environmental setting was always kept the same for all the recordings.

Subsequently, the collected video clips were validated by an independent group of three female evaluators from the University of Modena and Reggio Emilia. The evaluators, who were blind to the real nature of each video clip, had to watch the video clips and rate, on a scale from zero to ten, both IE and IP. Starting from this validation, 108 video clips (belonging to GP, FP, SP, and N categories) were selected, namely, the ones that received the most consistent ratings from the independent evaluators. The IE mean and the IP mean of the selected video clips, divided into the four above-mentioned categories, are represented in Table 1.

### 2.5. fMRI Data Acquisition

Functional MRI data were acquired with a Philips Achieva MRI system (Philips, Amsterdam, The Netherlands) at 3T and a BOLD (blood oxygenation level-dependent) sensitive gradient-echo echo-planar sequence [repetition time (TR): 2000 ms; echo time (TE): 30 ms; field of view: 240 mm; 80 × 80 matrix; 35 transverse slices, 3 mm each with a 1 mm gap]. A high-resolution T1-weighted anatomical image was also acquired for each subject to allow anatomical localization and spatial standardization (TR: 9.9 ms; TE: 4.6 ms; 170 sagittal slices; voxel size: 1 mm × 1 mm × 1 mm).

### 2.6. Behavioral Data Analysis

To perform behavioral analysis, RStudio (version 2024) and TIBCO Statistica, version 14.0.1 (2020) were used.

The questionnaires were scored for each participant, and the mean score was calculated for each questionnaire.

Regarding *a posteriori* video clips ratings, IE and IP were computed for each video clip category (GP, FP, SP, and N). Analyses of data distribution using the Shapiro-Wilk test showed that IE and IP were not normally distributed; therefore, a non-parametric ANOVA (Friedman’s test) with category as a within factor was conducted. Post-hoc analyses were performed using Wilcoxon signed-rank tests, with the Bonferroni correction applied for multiple comparisons to control for the probability of making at least one Type I error across all tests.

### 2.7. fMRI Data Analysis

The Matlab R2020a and SPM12 (Wellcome Department of Imaging Neuroscience, London, UK) software were used for fMRI data analysis.

For each participant, all functional volumes were corrected for slice timing, realigned to the first volume acquired, co-registered with the anatomical image, normalized to the MNI (Montreal Neurological Institute) template implemented in SPM12, and smoothed with a 6 mm × 6 mm × 8 mm full-width at half-maximum Gaussian kernel.

At the single-subject level, the four conditions (GP, FP, SP, and N) were modeled by convolving the respective stimulus timing vectors with the standard hemodynamic response function. Condition effects were estimated using a general linear model (GLM) framework, and region-specific effects were investigated with linear contrasts comparing the four experimental conditions. For all the subjects, the stimuli entered in each condition were the same, following the *a priori* categorization that derived from the preliminary validation of the video clips (see above). Head-motion parameters (translations and rotations) were entered as nuisance variables. Then, group-level random effects analyses were performed by entering the individual contrast images corresponding to the effects of interest into separate one-sample t-tests. Moreover, we conducted a conjunction analysis on genuine pain vs. neutral and fake pain vs. neutral to observe common activations with the same statistical values in order to evaluate regions involved in the processing of clearly visible facial expressions of pain.

Parametric analyses were performed to map regions whose activity was related to IP and IE, using the *a posteriori* categorization that we established by considering the IP and IE ratings, respectively, given by participants after the scanning session. In order to form the categories, we followed the rules illustrated in Table 2. We assigned the N category to the videos that received a score of 0 for both IE and IP. We then assigned the ‘Other’ category to those videos that had non-zero IE and 0 IP scores, where participants had indicated another type of perceived emotion (e.g., surprise, anger). Finally, we kept the original category for those videos that received non-zero scores for both IE and IP. On average, the N category comprised 18 stimuli, the GP category 33.9 stimuli, the FP category 20.1 stimuli, the SP category 20.9 stimuli, and the “Other” category 13.5 stimuli.

At the single-subject level, the five conditions (GP, FP, SP, N, and Other) were modeled by convolving the respective stimulus timing vectors and the respective IE/IP ratings with the standard hemodynamic response function. Head-motion parameters (translations and rotations) were entered as nuisance variables. At the group level, random effects analyses were performed by entering the individual contrast images corresponding to the effects of interest into separate one-sample *t*-tests. The stimuli were recategorized according to the participants’ responses, and for each stimulus, the corresponding IE/IP value was included in the analysis with the aim of separately evaluating the parametric effect on the different stimulus categories. However, the analysis of the individual categories did not yield results above the threshold, possibly because the variability in the estimates provided by the participants for stimuli belonging to each single category was too small. Therefore, we decided to use a single contrast that combined the parametric effect across all categories.

Furthermore, the personality questionnaire scores and the IA results were used to perform correlation analyses with functional brain activity in the various conditions/contrasts.

For all the above-mentioned analyses, a double statistical threshold (single-voxel statistics and spatial extent) was used to achieve a combined experiment-wise (i.e., corrected for multiple comparisons) significance level of α < 0.05, as computed by the 3dClustSim AFNI routine with the “-acf ” option (AFNI version 24.2.06).

## 3. Results

### 3.1. Behavioral Results

#### 3.1.1. Questionnaires and Interoception Assessment

The mean of EQ was 46.1 with a SD of 8.1; the mean of IRI was 87.9 with a SD of 8.9; the mean of TAS-20 was 50.2 with a SD of 8.4; the mean of PCS was 20.1 with a SD of 6.6. The mean of IA was 0.41 with a SD of 0.28.

#### 3.1.2. IE and IP Ratings

Regarding IE, fake stimuli received the highest ratings (M = 5.2, SD ± 2.2), followed by genuine (M = 4.2, SD ± 2.6), suppressed (M = 2, SD ± 1.8), and neutral (M = 0.5, SD ± 0.8). Concerning IP, genuine stimuli received the highest ratings (M = 4, SD ± 2.8), followed by fake (M = 3.5, SD ± 3), suppressed (M = 3.2, SD ± 2.5), and neutral (M = 1.5, SD ± 2).

**Figure 2:**
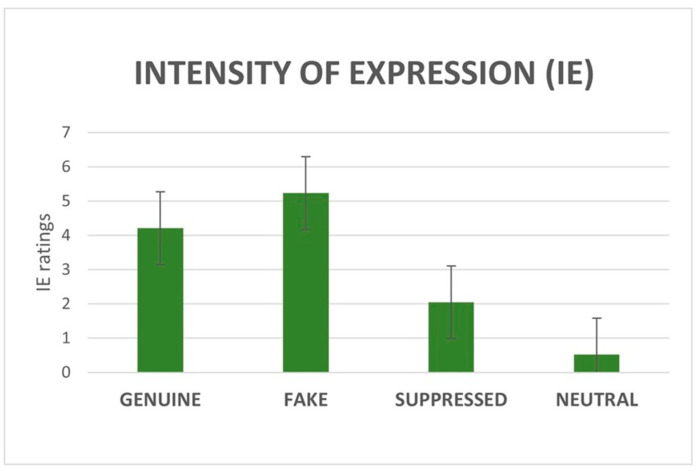
Main effect of category on IE ratings. All comparisons were statistically significant (*p* < 0.001). Error bars depict standard deviation (SD).

The non-parametric ANOVA (Friedman’s test) on IE ratings revealed a significant effect of category (χ^2^(3) = 1159.18, *p* < 0.001). Post-hoc tests revealed a significant difference between all the categories (*p* < 0.001; Figure 2).

**Figure 3:**
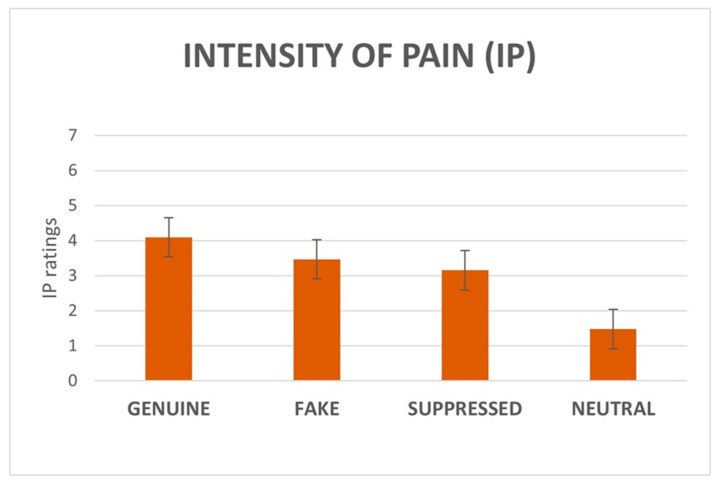
Main effect of category on IP ratings. All comparisons were statistically significant (*p* < 0.001), apart from the fake vs suppressed comparison (*p* < 0.05, does not resist Bonferroni correction). Error bars depict standard deviation (SD).

The non-parametric ANOVA (Friedman’s test) on IP ratings revealed a significant effect of category (χ^2^(3) = 347.03, *p* < 0.001). Post-hoc tests revealed significant differences among all the categories with *p* < 0.001, except for the difference between fake and suppressed that gave a *p* < 0.05. However, after applying the Bonferroni correction, the difference between fake and suppressed did not resist (Figure 3).

### 3.2. fMRI Results

#### 3.2.1. fMRI Results with *a Priori* Categorization

The following are the most relevant results of the *a priori* categorization analysis.

**Figure 4:**
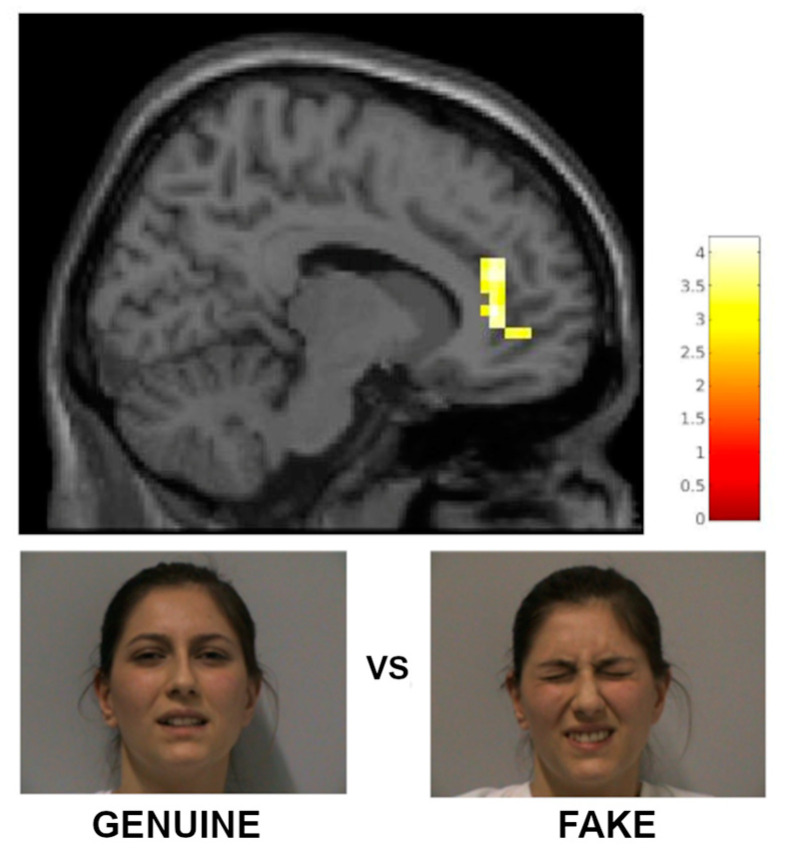
Regions of increased signal for the contrast GP vs. FP (x = 12). L = left; cluster-size threshold k > 46 voxels.

Greater BOLD response for the observation of genuine pain compared to fake pain (GP vs. FP) was observed in the pregenual anterior cingulate cortex (pACC; [28]), bilaterally (Table 3, Figure 4).

The contrast between genuine pain and suppressed pain (GP vs. SP) revealed areas of significant BOLD signal changes in a wide range of cortical regions, including the left cerebellum, bilateral superior, middle and inferior temporal gyrus, right fusiform gyrus, bilateral middle occipital gyrus, right inferior parietal lobule, right cuneus, and right insula (see Table 3).

The results of the conjunction analysis performed on the contrasts GP vs. N and FP vs. N revealed greater BOLD response in bilateral superior, middle, and inferior temporal gyrus, bilateral middle occipital gyrus, right inferior parietal lobule, right insula, right temporal pole, left supramarginal gyrus, right middle frontal gyrus and bilateral inferior frontal gyrus, left cerebellum, right inferior occipital gyrus, and right fusiform gyrus (Table 4).

#### 3.2.2. Parametric Analysis with *a Posteriori* Categorization

**Figure 5:**
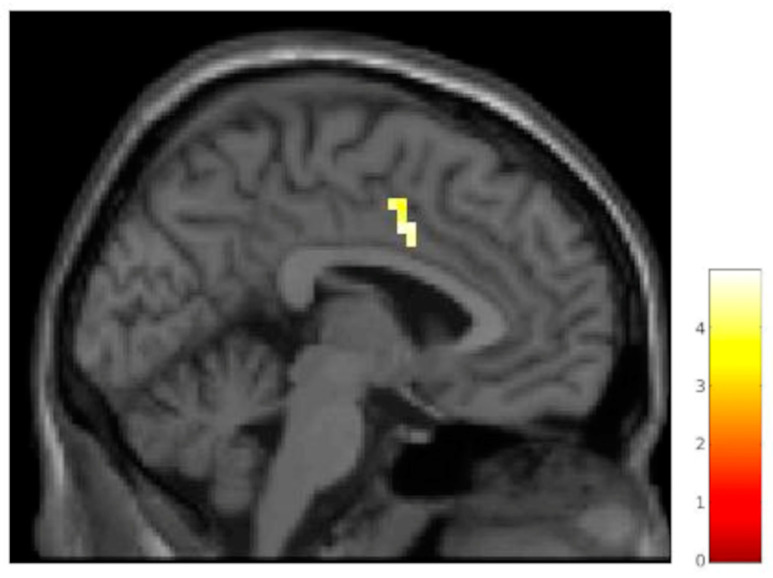
Regions whose activity is related to IP ratings (x = −3). L = left; cluster size threshold k > 9.

A significant parametric relationship was found between the IP ratings provided by the participants for each video clip and brain activity in the anterior mid-cingulate cortex (aMCC; Table 5, Figure 5).

Parametric analysis with IE ratings did not result in any significant activity.

#### 3.2.3. Correlations with Questionnaires and Interoception

No significant correlations were found in the most relevant contrasts of the *a priori* categorization analysis presented above for the other questionnaires, nor for the IA assessment results.

## 4. Discussion

The present study aimed to explore the behavioral and neural correlates of the observation of genuine, fake, and suppressed facial expressions of pain. Specifically, we sought to determine whether the brain can unveil the truth in pain expressions. To this end, we asked a group of women to undergo a functional magnetic resonance imaging (fMRI) task during which video clips depicting genuine pain (GP), fake pain (FP), suppressed pain (SP), and neutral (N) facial expressions were presented. After the scanning session, participants reviewed the video clips and provided *a posteriori* ratings for each video. As far as we are aware, this is the first study to use authentic video clips of facial expressions, where genuine and suppressed pain expressions were derived from actual nociceptive stimuli and deceptive expressions were deliberately simulated by the volunteers. Moreover, in order to minimize potential biases and emphasize automatic neural responses to different categories of pain expressions, we decided to employ an implicit task (gender discrimination) during the fMRI paradigm. In addition, during the post-scan video review, we employed an explicit but indirect task (i.e., subjects had not been told that some of the expressions they observed were genuine, while others were fake or suppressed, and they were not asked to directly recognize who was pretending). In this indirect task, participants rated the video clips according to the intensity of the facial expression (IE) and the intensity of the pain (IP) they observed. Overall, our findings indicate that participants were, on average, able to differentiate between genuine, fake, and suppressed pain through the indirect task. On the neural level, pregenual anterior and middle cingulate cortex activity appears to be crucial in unveiling genuine pain and coding the intensity of pain observed in others.

### 4.1. Behavioral Findings

Our participants assigned average higher IP ratings to genuine pain facial expressions compared to fake, suppressed, and neutral ones. On the contrary, they assigned average higher IE ratings to fake pain facial expressions compared to genuine, suppressed, and neutral ones. As it is known from the literature that simulated expressions tend to be more pronounced in terms of expressiveness, with the typical components of genuine pain—such as brow lowering and mouth opening—being displayed with greater intensity and for longer durations compared to authentic expressions [12], both these results suggest that subjects were indirectly able to distinguish genuine from simulated expressions of pain. This ability could also be enhanced by the fact that simulated facial expressions often include incongruent non-pain-related actions, conveying emotions such as shame, guilt, or happiness [12,15].

It is also worth noting that suppressed pain expressions showed lower IE ratings than genuine and simulated expressions and higher IE ratings than neutral expressions. These results confirm previous research demonstrating that suppressed pain facial expressions are less intense, but also that they present some residual facial activity (e.g., micro-expressions, such as brow lowering and mouth opening) [12,15]. These micro-expressions might be precisely what lead participants to attribute higher IP ratings to suppressed expressions compared to neutral expressions: it appears that participants were only partly deceived by the effort made by the models in the video clips to mask the pain they actually felt.

Our behavioral results support the hypothesis that asking individuals to rate the expressivity and the pain observed in others allow the recognition of the authenticity of pain facial expressions. This kind of task could be categorized as an indirect task according to the dissociation between direct vs. indirect measures, as it does not directly ask participants whether the models are lying [3,13,15,18,19,20].

### 4.2. Functional Imaging Findings

According to the dissociation between explicit and implicit emotional processing tasks [22,50], our fMRI protocol can be categorized as an implicit task. Indeed, participants performed a non-emotional task (gender discrimination) with consciously visible emotional stimuli (i.e., pain facial expressions).

Several studies consistently demonstrate that a well-known set of brain regions (“affective” areas of the Pain Matrix) is involved in the observation of facial expressions of pain [4,5,31,32]. Within these regions, the anterior mid-cingulate cortex (aMCC) and the anterior insula (AI) play key roles [29]. It is worth noting that the cingulate cortex nomenclature has been quite controversial in the past few decades; therefore, the same region (aMCC) is also variably named in the literature (e.g., the anterior cingulate cortex, ACC, or dorsal ACC; see, for instance, [31]). Here, we follow Vogt’s classification of the cingulate cortex [28].

The parametric analysis with *a posteriori* categorization revealed that aMCC activity is related to IP ratings, i.e., the greater the IP score, the greater the activity of aMCC. This region is commonly considered part of the affective network elaborating physical pain [26], and its activity correlates with the intensity of perceived physical pain [30]. aMCC was also found to be activated in various studies comparing the observation of painful vs. non-painful facial expressions [4,5,31,32]. The present finding suggests that aMCC might be the brain structure mainly involved in the “measure” of pain, and therefore in the discrimination between real and fake pain. Interestingly, Budell and colleagues [32] indicated this region, along with several others, as correlating with the amount of pain judged to be present in the observed facial expressions; however, they used as stimuli only pain expressions produced by actors presented as real. A previous study on genuine/deceptive pain expressions [33] proposed that genuine pain, compared to simulated pain, selectively activates aMCC, AI, and right supramarginal gyrus (rSMG), then focused on the interplay between AI and rSMG, rather than on aMCC. However, it is worth noting that the study by Zhao et al. [33] presents several differences compared to our experimental protocol: first, participants were explicitly informed that they would observe deceptive expressions; second, they were also explicitly instructed to recreate the feelings of the models as vividly and intensely as possible; therefore, these instructions might have introduced some significant biases. Moreover, all their videos depicted faces of actors intentionally producing facial expressions of pain; therefore, even the expressions of “genuine” pain were actually simulated.

Our results also show that the pregenual anterior cingulate cortex (pACC) is significantly more activated when observing genuine pain expressions [51,52] compared to fake ones. Some studies have shown activation of the subgenual and perigenual portions of the ACC (corresponding to pACC in Vogt’s classification [28]) for nociceptive stimuli [53], as well as saline injections or visceral distention [54], although not as consistently as for other regions. In fact, the anterior regions of the cingulate cortex are not listed in a recent meta-analysis on the neural representation of acute pain [26]. Interestingly, the pACC showed increased signal when observing negative affective images of body parts [55] when comparing self vs. others’ faces in a study about pain expressions [5], but decreased signal both during and in the anticipation of pain acute perception [56]. Furthermore, the pACC has connections with the orbitofrontal cortex, periacqueductal grey, and autonomic centers and is rich in opioids; overall, this region has been consistently implicated in endogenous pain control, including placebo [51]. The increased signal we found in the pACC may be related to the activation of endogenous control systems, possibly related to an empathic response, which is higher when observing genuine than fake pain expressions.

Additionally, the observation of authentic facial expressions of pain, as compared to suppressed ones (GP vs. SP), more intensely activated a bilateral array of cortical areas located in the temporal, occipital, and insular cortices. These brain regions, and particularly AI, are related both to actual pain perception and to the observation of pain [5,24], as well as to facial processing according to the Haxby and Gobbini model [57,58] Indeed, several studies consistently demonstrated that AI is engaged in processing the distressing aspects of pain [29], to the point that its activation correlates with the questionnaire responses regarding the intensity of perceived pain [30]. On the other hand, the superior temporal gyrus and right fusiform gyrus contribute to both the visual processing of facial expressions (i.e., core system) and the emotional interpretation of their features (i.e., extended system) [57,58]. A possible explanation for why suppressed expressions failed to elicit such brain activations is that inhibited expressions do not actually present a sufficient amount of expressive cues to activate the network. This is certainly consistent with the behavioral data; indeed, IE ratings of suppressed pain expressions are higher than neutral, but lower than all the other ones. Previous studies on emotional body expressions demonstrated that perceiving negative (especially fearful) body expressions significantly activates dorsal stream structures involved in action preparation, with a central role of the parietal cortex [59,60,61,62]. In addition, the identification of facial expressions of emotions was shown to elicit activations in the inferior parietal lobe (IPL; [63]). Consistently, IPL activation was observed during the evaluation of dynamic expressive faces compared to gender evaluation [64], further highlighting the IPL’s role in processing emotionally salient facial cues. Nevertheless, the neural representations of categorical valence (positive, neutral, and negative) were identified within several regions, including IPL, as well as the precuneus, bilateral medial prefrontal cortex (MPFC), left superior temporal sulcus (STS)/postcentral gyrus, right STS/middle frontal gyrus (MFG), and thalamus [65]. Furthermore, emotion-specific but stimulus category-independent neural representations were observed in the left postcentral gyrus, left IPL, and right STS [66].

Buhle and colleagues [67] employed an fMRI study and examined eight independent datasets (218 total subjects) to investigate the neural overlap between the experience of pain and the processing of negative emotions. Even if the study primarily examined the role of the periaqueductal gray, the authors found that other regions were involved in both conditions, including the right inferior parietal lobule and bilateral cuneus. The inferior parietal lobule is located near the secondary somatosensory cortex, and it is linked to both the direct experience of pain and the imagination or observation of pain from both the body and the face [32,68,69]. Cuneus activity is primarily associated with visual processing, and heightened attention to emotional images may have enhanced visual processing for negative compared to neutral stimuli. Interestingly, cuneus activity has also been linked to the affective dimension of pain [70,71]. These findings suggest that the cuneus may play a broader role in processing visual aversive stimuli, similarly to what we observed in our study for genuine and fake emotional expressions.

Although a distinction between genuine and deceptive pain expressions was observed at the level of the pACC, it is noteworthy that the conjunction analysis (GP vs. N and FP vs. N) showed that both types of expressions activate a widespread common network, similar to the one just mentioned, which also included other cortical regions such as bilateral inferior frontal gyrus (IFG) and left supramarginal gyrus (lSMG). IFG is hypothesized to represent a fundamental region within the human mirror neuron system [72]. This, in turn, may represent a mechanism of shared representations proposed to underlie empathy [73], and could be relevant for the process of mentalizing similarity between oneself and the target that one is observing [74]. Additionally, recent findings indicate that the rIFG could play a role in interpreting pain through visual information (encompassing not only facial expressions but also sensory cues), facilitating the observer’s ability to infer another individual’s state and forming the neural foundation for empathy toward pain [32,75]. Consistent with this evidence, our study observed significant activations of the right IFG when facial expressions of pain, whether genuine or simulated, were presented, as compared to neutral facial expressions. This can be explained by a heightened empathic resonance elicited by viewing these images, in contrast to the neutral facial expressions. Recent evidence suggests that the right supramarginal gyrus (SMG) could play a pivotal role in distinguishing self from others affective state [76,77], and in the interaction with affective regions, such as AI, in order to shape the behavioral response to observed pain [78,79]. Recent evidence suggests that during genuine pain scenarios, this modulatory effect could prevent empathic over-arousal and enable appropriate social responses [33]. In line with the literature, our result in the GP vs. N and FP vs. N conjunction shows a greater BOLD response in the left SMG when participants see a dynamic facial expression of pain compared to when neutral facial expressions are presented. This may be due to a greater empathic resonance elicited by facial expressions of pain compared to neutral ones, reinforcing the aforementioned role of the SMG in empathic responses to pain scenarios.

Regarding the cerebellum, several studies have reported its involvement in physical pain perception, with activity centered in its left hemisphere, posterior lobe (see meta-analyses [25,26]). The left posterior activations we found during the observation of genuine pain expressions compared to suppressed ones, and in both genuine and fake expressions, substantially overlap with activation found in studies comparing painful with neutral expressions [31]. Interestingly, previous studies showed widespread posterior cerebellar activation during implicit emotion (not pain) processing [23,50,80].

Previous studies focusing on the neural correlates of implicit and explicit processing of emotional facial expressions have shown that both tasks were able to activate a common network of brain regions, including the occipital and temporal lobes, IFG, and cerebellum, engaged in high-valence stimuli processing. At the same time, some neural distinctions between implicit and explicit emotion processing are proposed. Explicit processing seemed to activate to a greater extent the middle temporal gyrus and the IFG [23,50], whereas implicit processing involved a greater activation of the amygdala [23]. Our results align with previous findings regarding negative emotions; indeed, using an implicit task, we observed activations in occipital and temporal gyri, IFG, and cerebellum during the observation of highly expressive pain facial expressions (GP vs. N and FP vs. N conjunction). The presence of these activations in response to pain expressions, as well as other basic emotions, provides additional evidence supporting the notion that pain fits the paradigms of emotions. We did not demonstrate an enhanced amygdala activation, but this is in line with a study by Scheuerecker et al. [50].

### 4.3. Limitations and Future Directions

The sample consisted exclusively of women, which may limit the generalizability of the findings to the general population. Additionally, the sample consisted only of young students, which ensured homogeneity; however, it certainly limited the results’ generalizability.

Future directions of research may include extending the experimental paradigm to male participants in order to assess potential gender differences in emotional processing. Moreover, future studies could benefit from a more diverse age range to explore potential age-related differences in the neural mechanisms under investigation.

These results may have significant implications in clinical practice, as they support the idea that caregivers and healthcare professionals could be trained to use these indirect/implicit processes to unveil the truth in others’ pain. Indeed, the assessment of pain is essential to provide appropriate care to individuals in need. A significant concern for the healthcare community involves vulnerable populations who are unable to communicate or express their pain directly. These groups include infants, young children, individuals with mental illnesses, and the elderly. In such cases, caregivers or family members typically rely on observing behavioral or physiological responses to infer the presence or absence of pain. In some cases, observers may lack the necessary training or may be influenced by personal biases, leading to inadequate or inaccurate assessments of the patient’s pain experience [81,82]. Furthermore, social and interpersonal dynamics can affect not only the expression and perception of pain but also the judgments made by those evaluating it [83]. For these reasons, understanding and quantifying the ability to recognize facial expressions of pain represents a crucial first step in developing specific training programs.

## 5. Conclusions

This study provides valuable insights into both the behavioral and neural mechanisms underlying the recognition of pain expressions. From a behavioral perspective, participants demonstrated an ability to distinguish genuine pain from deceptive expressions, while suppressed expressions were recognized as less intense. Regarding the functional perspective, fMRI findings indicate that a specific brain region, namely the pACC, is more activated by genuine pain facial expressions compared to fake ones. Moreover, the aMCC appears to play a crucial role in evaluating the intensity of pain, with its activation correlating with the perceived pain. These findings contribute to the broader understanding of emotional recognition processing, offering a deeper perspective on the neural dynamics involved in distinguishing genuine from deceptive pain in others.

## Figures and Tables

**Table 1:** Validation of the stimuli used in the task. Mean (M) and standard deviation (SD) for intensity of expression (IE) and intensity of pain (IP) provided by three independent evaluators for the selected video clips divided into the four categories (genuine, fake, suppressed, and neutral).

	Genuine		Fake		Suppressed		Neutral	
	IE	IP	IE	IP	IE	IP	IE	IP
**M**	3.4	2.4	3.2	0.04	1.2	2.5	0	0
**SD**	1.1	1.3	0.4	0.2	0.4	0.7	0	0

**Table 2:** Rules used for the *a posteriori* categorization. From the left column: Intensity of expression (IE), intensity of pain (IP), and the condition in which the stimuli are inserted.

IE	IP	Condition
0	0	Neutral
≠0	0	Other
≠0	≠0	Real/Fake/Suppressed (based on the *a priori* categorization)

**Table 3:** Regions of increased signal for the contrast GP vs. FP and GP vs. SP. BA = Brodmann Area; l = left; r = right; cluster-size threshold k > 46 voxels and k > 62 voxels, respectively, to reach the combined alfa < 0.05.

	BA	Side	Cluster	Voxel Level	MNI Coordinates			Talairach Coordinates		
Brain Areas			k	Z_E_	x	y	z	x	y	z
**GP vs. FP**										
pregenual anterior cingulate cortex	32	r/l	125	3.59	12	38	6	12	38	12
**GP vs. SP**										
Cerebellum		l	67	5.01	−15	−73	−46	−15	−73	−35
				3.72	−18	−67	−30	−18	−66	−22
				2.95	−21	−79	−38	−21	−78	−28
Middle Temporal Gyrus, Superior Temporal Gyrus, Inferior Temporal Gyrus, Inferior Parietal Lobule, Fusiform Gyrus, Middle Occipital Gyrus, Insula, Cuneus	21, 22, 37, 40 19, 17	r	579	4.74	48	−37	2	48	−36	4
				4.54	51	−73	2	50	−71	5
				4.50	57	−40	6	56	−38	7
Middle Temporal Gyrus, Superior Temporal Gyrus. Inferior Temporal Gyrus, Middle Occipital Gyrus.	22, 19	l	221	4.55	−54	−46	6	−53	−44	8
				3.99	−48	−70	6	−48	−68	9
				3.21	−39	−70	6	−39	−68	9

**Table 4:** Regions of increased signal obtained from the conjunction between the contrasts GP vs. N and FP vs. N. BA = Brodmann Area; l = left; r = right; *p* < 0.001 uncorrected, cluster-size threshold k > 0 voxels. For the visualization the extension k > 9 was selected.

	BA	Side	Cluster	Voxel Level	MNI Coordinates			Talairach Coordinates		
Brain Areas			k	Z_E_	x	y	z	x	y	z
Middle Temporal Gyrus, Superior Temporal Gyrus, Inferior Temporal Gyrus, Middle Occipital Gyrus, Inferior Parietal Lobule, Insula	21, 22, 20, 19, 39	r	599	6.98	51	−40	6	50	−38	7
				6.06	48	−76	−2	48	−74	2
				5.84	45	−31	−6	45	−30	−4
Middle Temporal Gyrus, Superior Temporal Gyrus, Temporal Pole	21, 38	r	46	5.95	54	8	−22	53	7	−19
Middle Temporal Gyrus, Superior Temporal Gyrus, Inferior Temporal Gyrus, Middle Occipital Gyrus, Supramarginal Gyrus	22, 37, 19	l	360	5.60	−51	−73	6	−50	−70	9
				5.58	−51	−64	6	−50	−62	9
				5.30	−57	−55	6	−56	−53	8
Middle Frontal Gyrus	46	r	33	5.43	48	2	42	48	4	38
Inferior Frontal Gyrus, Insula	47	r	64	4.85	45	26	−2	45	25	−3
Inferior Frontal Gyrus	47	l	70	4.80	−48	20	−6	−48	19	−6
				3.94	−39	26	−2	−39	25	−3
Cerebellum		l	14	4.14	−12	−76	−46	−12	−76	−35
				3.53	−18	−82	−46	−18	−81	−35
Middle Occipital Gyrus, Inferior Occipital Gyrus, Fusiform Gyrus	18, 37	r	41	4.03	27	−85	−6	27	−83	−1
				3.60	27	−91	10	27	−88	14
Fusiform Gyrus	37	r	16	3.96	42	−43	−22	42	−43	−16

**Table 5:** Regions whose activity is related to IP ratings. BA = Brodmann Area; l = left; cluster size threshold k > 9, to reach the combined alfa < 0.05.

	BA	Side	Cluster	Voxel Level	MNI Coordinates			Talairach Coordinates		
Brain Areas			k	Z_E_	x	y	z	x	y	z
Mid-cingulate cortex	24	l	15	4.68	−3	2	38	−3	4	35

## Data Availability

The data presented in this study are available on request from the corresponding author. Data is unavailable publicly due to privacy and ethical restrictions.

## Abbreviations

The following abbreviations are used in this manuscript:

ACC	Anterior cingulate cortex
AI	Anterior insula
aMCC	Anterior mid-cingulate cortex
BOLD	Blood oxygenation level-dependent
EQ	Empathy quotient
fMRI	Functional magnetic resonance imaging
FP	Fake pain
GLM	General linear model
GP	Genuine pain
IA	Interoceptive accuracy
IASP	International association for the study of pain
IE	Intensity of the facial expression
IFG	Inferior frontal gyrus
IP	Intensity of the pain
IPL	Inferior parietal lobe
IRI	Interpersonal reactivity index
M	Mean
MFG	Middle frontal gyrus
MNI	Montreal neurological institute
MPFC	Medial prefrontal cortex
N	Neutral
NRS	Numerical rating scale
pACC	Pregenual anterior cingulate cortex
PCS	Pain catastrophizing scale
SD	Standard deviation
SMG	Supramarginal gyrus
SP	Suppressed pain
SPM	Statistical parametric mapping
STS	Superior temporal sulcus
TAS-20	Toronto alexithymia scale 20
TE	Echo time
TR	Repetition time
WS	Warning signal
